# Dosimetric Study of Automatic Brain Metastases Planning in Comparison with Conventional Multi-Isocenter Dynamic Conformal Arc Therapy and Gamma Knife Radiosurgery for Multiple Brain Metastases

**DOI:** 10.7759/cureus.882

**Published:** 2016-11-15

**Authors:** Yoshimasa Mori, Naoki Kaneda, Masahiro Hagiwara, Tuneo Ishiguchi

**Affiliations:** 1 Department of Radiology and Radiation Oncology, Aichi Medical University; 2 Department of Radiology and Radiation Oncology, Archie Medical University; 3 Nagoya Radiosurgery Center, Nagoya Kyoritsu Hospital

**Keywords:** brain, metastasis, stereotactic radiosurgery, multiple lesions, gamma knife, linear accelerator, dynamic conformal arc, automatic brain metastases planning element, iplan, dose gradient

## Abstract

**Objective:**

The efficacy of stereotactic radiosurgery (SRS) using Gamma Knife (GK) (Elekta, Tokyo) is well known. Recently, Automatic Brain Metastases Planning (ABMP) Element (BrainLAB, Tokyo) for a LINAC-based radiation system was commercially released. It covers multiple off-isocenter targets simultaneously inside a multi-leaf collimator field and enables SRS / stereotactic radiotherapy (SRT) with a single group of LINAC-based dynamic conformal multi-arcs (DCA) for multiple brain metastases. In this study, dose planning of ABMP (ABMP-single isocenter DCA (ABMP-SIDCA)) for SRS of small multiple brain metastases was evaluated in comparison with those of conventional multi-isocenter DCA (MIDCA-SRS) (iPlan, BrainLAB, Tokyo) and GK-SRS (GKRS).

**Methods:**

Simulation planning was performed with ABMP-SIDCA and GKRS in the two cases of multiple small brain metastases (nine tumors in both), which had been originally treated with iPlan-MIDCA. First, a dosimetric comparison was done between ABMP-SIDCA and iPlan-MIDCA in the same setting of planning target volume (PTV) margin and D95 (dose covering 95% of PTV volume). Second, dosimetry of GKRS with a margin dose of 20 Gy was compared with that of ABMP-SIDCA in the setting of PTV margin of 0, 1 mm, and 2 mm, and D95=100% dose (20 Gy).

**Results:**

First, the maximum dose of PTV and minimum dose of gross tumor volume (GTV) were significantly greater in ABMP-SIDCA than in iPlan-MIDCA. Conformity index (CI, 1/Paddick’s CI) and gradient index (GI, V (half of prescription dose) / V (prescription dose)) in ABMP-SIDCA were comparable with those of iPlan-MIDCA. Second, PIV (prescription isodose volume) of GKRS was consistent with that of 1 mm margin - ABMP-SIDCA plan in Case 1 and that of no-margin ABMP-SIDCA plan in Case 2. Considering the dose gradient, the mean of V (half of prescription dose) of ABMP-SIDCA was not broad, comparable to GKRS, in either Case 1 or 2.

**Conclusions:**

The conformity and dose gradient with ABMP-SIDCA were as good as those of conventional MIDCA for each lesion. If the conditions of the LINAC system permit a minimal PTV margin (1 mm or less), ABMP-SIDCA might provide excellent dose fall-off comparable with that of GKRS thereby enabling a short treatment time.

## Introduction

Gamma Knife (GK) (Elekta, Tokyo) stereotactic radiosurgery (SRS) (GKRS) can treat multiple small brain metastases easily with multi-isocenter planning [1]. Even if the brain metastases have a radio-resistant histological nature such as those from melanoma and renal cell carcinoma, they can be treated effectively by GKRS [2-3]. As shown in most of the formerly published studies, GK provides good conformity and an excellent dose gradient [4], but shows less homogeneity, which may be an advantage for tumor ablation as well [5]. However, the treatment time (beam-on-time) in GKRS will be long in the case of numerous brain metastatic lesions, though shorter in most cases than that possible with CyberKnife [6].

**Figure 1:**
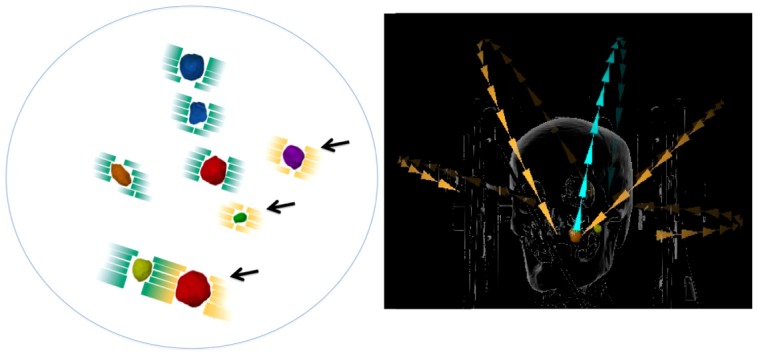
Automatic Brain Metastases Planning (ABMP)-single isocenter dynamic conformal arc (ABMP-DCA) In this case, all eight tumors are covered with a field of multi-leaf collimator (Left). Three tumors (*arrows*) are irradiated by ‘return’ arc in this case. All tumors are irradiated by one multi-arc group. Each tumor is targeted by some of the 10 arcs, five arcs by ‘go’ and ‘return,’ in this case (Right).

Linear accelerator (LINAC)-based dynamic conformal multi-arc (DCA) SRS and stereotactic radiotherapy (SRT) are also effective for brain metastases with a small number of tumors [7]. Conventional DCA SRS/SRT, for example, planned with iPlan (BrainLAB, Tokyo), needs a set of DCA for each individual lesion, and the treatment time would be long as well. Recently, Automatic Brain Metastases Planning (ABMP) Element (BrainLAB, Tokyo) was commercially released. It covers multiple off-isocenter targets simultaneously inside a series of multi-leaf collimator fields and enables SRS/SRT with a single group of LINAC-based DCA for multiple brain metastases. In ABMP-DCA, multiple brain metastases up to 10 tumors are covered in a micro-multi-leaf collimator field. Figure 1 shows an example of multiple brain tumor SRS by ABMP. All tumors are irradiated by one multi-arc group (Figure 1, Right). Each tumor is targeted by some of the 10 arcs, five arcs by ‘go’ and ‘return’. Three tumors (arrows) are irradiated by ‘return’ arc in this case (Figure 1, Left). In this way ABMP facilitates a shorter treatment time.

In this study, dosimetry of ABMP (ABMP-single isocenter DCA (ABMP-SIDCA)) for SRS of multiple small brain metastases was evaluated in comparison with that of conventional multi-isocenter DCA (iPlan-MIDCA) and GKRS.

## Materials and methods

The Research Ethics Boards of Aichi Medical University (No.2015-H332) and Nagoya Kyoritsu Hospital approved this study. Informed consent was waived for this study. Simulation planning was performed with ABMP-SIDCA, and GKRS was made in two cases with multiple small brain metastases, which had originally been treated with iPlan-MIDCA. Both cases had nine metastatic brain tumors.

### Case 1

Case 1 was a 71-year-old female with multiple brain metastases from breast carcinoma. All nine brain tumors were treated by conventional iPlan-MIDCA. A planning target volume (PTV) margin of 2 mm was added. A leaf margin of 1 mm was adopted. D (95%) (dose to 95% volume of PTV) was 95% dose (=19 Gy) of 20 Gy. In iPlan-MIDCA, nine tumors were treated with four DCAs each. A three-day treatment was done, in which three of the nine tumors were treated each day.

**Figure 2:**
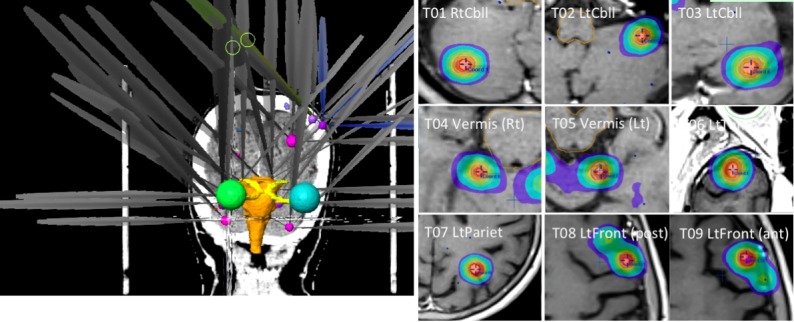
Conventional iPlan multi-isocenter DCA (iPlan-MIDCA)

Figure 2 (Left) displays all 36 arcs for nine tumors in conventional iPlan-MIDCA. The dose distribution for each tumor was displayed on the iPlan workstation (Figure 2, Right).

**Figure 3:**
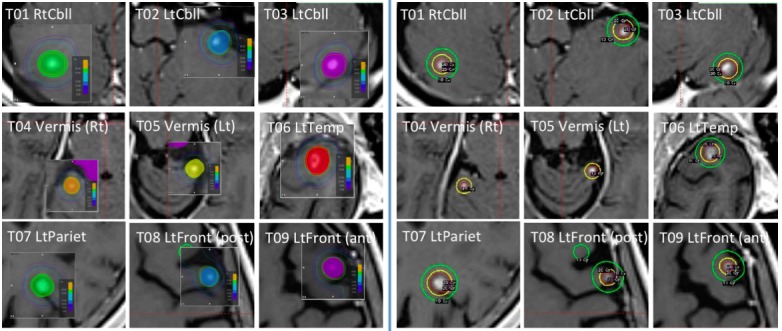
ABMP (left) and GammaPlan (right)

Simulation planning was performed with ABMP-SIDCA and GKRS. The dose distribution for each tumor was displayed on the ABMP workstation (Figure 3, Left) and the GammaPlan workstation (Figure 3, Right) in Case 1.

### Case 2

Case 2 was a 76-year-old female with multiple brain metastases from papillary thyroid carcinoma. All nine tumors were treated by iPlan-MIDCA. Each tumor was treated with four arcs. A PTV margin of 1 mm was added. A leaf margin of 1 mm was adopted. D (95%) was 100% dose of 22 Gy. A three-day treatment was done, in which three tumors were treated each day. In this case we gave a greater dose to the lesions than in Case 1 because thyroid carcinoma is thought to be relatively radio-resistant.

### Imaging protocol and version of radiation therapy planning workstations

To determine GTV (=clinical target volume (CTV)), contrast-enhanced magnetic resonance imaging (MRI) and computed tomography (CT) were acquired. A 3.0 tesla scanner (Siemens Skyra Ver.VE, Siemens, Tokyo) and a 16 detector CT (Aquilion/LB, Toshiba, Tokyo) were used. The references for dose calculation in treatment planning were the CT images in iPlan Image (version 4.1) and iPlan Dose (version 4.5.3) for iPlan-MIDCA and ABMP Elements (version 1.0) for ABMP-SIDCA. Pencil beam convolution algorithms were used in both. Leksell GammaPlan (LGP, version 10.1.1) treatment-planning workstation (Elekta, Tokyo) was used for GKRS. LGP adopted water reference dosimetry [8].

The CT parameters were as follows: kV peak, 120 kV; X-ray tube current, 420 mA; exposure time, 1500 msec; field of view (FOV), 512 x 512; slice thickness, 1.0 mm. The MRI was specified by 3D-VIBE (3-dimensional-volumetric interpolated breath-hold examination; TR, 10 msec; TE, 2.59 msec; FOV, 256; slice thickness, 0.7 mm; voxel size, 0.83 x 0.67 x 1.16 mm; scan time, 3:44) and 3D-SPACE (sampling perfection with application optimizes contrasts using different flip angle evolution; TR, 600 msec; TE, 22 msec; FOV, 256; slice thickness. 0.7 mm; voxel size, 0.83 x 0.67 x 1.16 mm; scan time, 6:25) with gadolinium (Gd) enhancement. In iPlan, contouring of GTV was made by 2D brush on axial images. In ABMP, contouring was performed by 3D brush. In LGP manual pen contouring was made on axial images.

### Dosimetric analysis

First, a dosimetric comparison was done between ABMP-SIDCA and iPan-MIDCA with the setting with PTV margin of 2 mm and D95=95% dose (19 Gy) in Case 1 and PTV margin of 1 mm and D95=100% dose (22 Gy) in Case 2. A leaf margin of 1 mm was adopted in iPlan-MIDCA in both cases. ABMP does not have the function of leaf margin selection. The indices of dosimetry were as follows: as for conformity index (CI), reverse of Paddick CI [9] was evaluated. Reverse of Paddick CI = (TVPIV)2 / (TV x PIV), where PIV is the prescription isodose volume, TVPIV is Target Volume covered by PIV, and TV is the target volume. CI was considered acceptable when smaller than two. The gradient index was calculated with the formula: GI = PIVhalf / PIV [10]. PIVhalf = Prescription isodose volume, at half the prescription isodose. The maximum dose in PTV and the minimum dose in GTV were also compared. Maximum doses to OARs (eyes, lenses, brainstem, and optic pathways) were also evaluated.

Second, dosimetry of GKRS (Figure 3B) was compared with that of ABMP-SIDCA with different PTV margins of 0, 1 mm, and 2 mm with the setting of D95=100% dose (20 Gy) in both Case 1 and Case 2. All nine tumors were treated with a one-isocenter plan in LGP in both cases. The percent isodose adopted as target margin in GKRS was 60% to 95% (median 85%) in Case 1 and 50% to 90% (median 80%) in Case 2. PIV (=V (prescription dose)) and V (half of prescription dose) were evaluated. The collected dosimetry data were analyzed using R version 2.14.2 (The R Foundation for Statistical Computing). The paired t-test was used to examine differences between indices of treatment plans. Differences with p < 0.05 were regarded as significant.

## Results

### Comparison between ABMP-SIDCA and iPlan-MIDCA

There was no significant difference in the means of PTV in either Case 1 (Table 1) or Case 2 (Table 3), though the manner of contouring differed, namely 3D brush contouring in ABMP and 2D brush contouring slice by slice in iPlan. Neither CI nor GI was significantly different between ABMP-SIDCA and iPlan-MIDCA. The maximum dose of PTV and minimum dose of GTV were significantly larger in ABMP-SIDCA than in iPlan-MIDCA. Both in Case 1 (Table 2) and in Case 2 (Table 4) the maximum doses to eyes and lenses were minimal in iPlan-MIDCA, because the arcs of iPlan-DCA were cut manually with the intention of sparing them.

**Table 1:** Comparison between iPlan-MIDCA and ABMP-SIDCA (Case 1) Prescription: PTV D95%=95% dose (19 Gy), Leaf margin on iPlan-MIDCA: 1mm No leaf margin function in ABMP-SIDCA. PTV=planned target volume (iPlan / ABMP), GTV=gross tumor volume, Vol.=volume (iPlan / ABMP) Cbll=cerebellar, Temp=temporal, Pariet=parietal, Front=frontal, rt=right, lt=left, v.=vermis CI=conformity index, GI=gradient index=V[half of prescription dose] / V[prescription dose]

	PTV (2mm margin)		No. of arcs		PTV Max.Dose(Gy)		GTV Min.Dose(Gy)		CI 1/Paddick’s CI		GI V[1/2PresD] / V[PresD]	
	Location	Vol.(ml)	iPlan	ABMP	iPlan	ABMP	iPlan	ABMP	iPlan	ABMP	iPlan	ABMP
1	Cbll (rt)	0.4 / 0.4	4	3	20.9	21.5	20.1	20.8	1.4	1.1	4.7	5.6
2	Cbll (lt)	0.2 / 0.3	4	4	20.9	22.1	20.0	21.3	1.6	1.2	5.1	5.1
3	Cbll (lt)	0.5 / 0.6	4	4	20.6	22.0	19.8	20.8	1.3	1.2	4.9	4.5
4	Cbll (v.)	0.2 / 0.2	4	4	20.9	22.2	20.1	21.4	1.6	1.2	6.0	5.4
5	Cbll (v.)	0.3 / 0.2	4	4	20.8	21.8	20.0	21.2	1.4	1.2	5.9	5.6
6	Temp (rt)	0.6 / 0.7	4	5	20.8	22.5	19.7	20.7	1.4	1.2	4.0	4.3
7	Pariet (rt)	0.7 / 0.5	4	3	21.6	21.9	19.7	20.9	1.5	1.1	3.4	5.1
8	Front (lt)	0.3 / 0.3	4	4	20.8	22.7	19.9	21.2	1.8	1.4	4.8	5.0
9	Front (lt)	0.3 / 0.3	4	3	22.0	23.0	20.1	21.3	1.8	2.9	4.8	5.4
	mean	0.4 / 0.4	4	3.8	21.0	22.2	19.9	21.1	1.53	1.36	4.84	5.12
		p=1			p <0.001		p <0.001		p=0.39		p=0.30	

**Table 2:** Comparison of OAR sparing between iPlan-MIDCA and ABMP-SIDCA (Case 1) Prescription: PTV D95%=95% dose (19 Gy) OPN-OPT=optic nerve-optic tract, rt=right, lt=left

	Max. dose (Gy)	
	iPlan	ABMP
Brainstem	10.8	8.1
Eye (lt)	1.0	2.7
Eye (rt)	0.9	3.8
Lens (lt)	0.7	1.6
Lens (Rt)	0.5	2.3
OPN-OPT (lt)	1.1	2.6
OPN-OPT (rt)	2.3	4.3

**Table 3:** Comparison between iPlan-MIDCA and ABMP-SIDCA (Case 2) Prescription: PTV D95%=100% dose (22 Gy), Leaf margin on iPlan-MIDCA: 1mm, No leaf margin function in ABMP-SIDCA, PTV=planned target volume (iPlan / ABMP), GTV=gross tumor volume, Vol.=volume (iPlan / ABMP), Cbll=cerebellar, Occip=occipital, Temp=temporal, Front=frontal, Pariet=parietal, rt=right, lt=left, v.=vermis CI=conformity index, GI=gradient index=V[half of prescription dose] / V[prescription dose]

	PTV (1mm margin)		No. of arcs		PTV Max.Dose(Gy)		GTV Min.Dose(Gy)		CI 1/Paddick’s CI		GI V[1/2PresD] / V[PresD]	
	Location	Vol.(ml)	iPlan	ABMP	iPlan	ABMP	iPlan	ABMP	iPlan	ABMP	iPlan	ABMP
1	Cbll (v.)	0.2 / 0.2	4	5	23.3	25.7	22.5	24.2	2.0	2.2	4.5	3.8
2	Occip (lt)	0.3 / 0.2	4	5	23.4	25.2	22.7	24.3	1.7	2.0	3.5	5.7
3	Temp (rt)	0.5 / 0.6	4	5	23.3	25.6	22.5	24.0	1.8	1.9	3.5	3.3
4	Temp (rt)	0.2 / 0.1	4	5	23.2	26.0	22.6	25.0	2.0	2.4	3.5	4.3
5	Occip (rt)	0.3 / 0.3	4	4	23.8	26.3	22.6	23.9	2.3	2.2	3.7	3.4
6	Front (lt)	1.7 / 2.1	4	4	23.6	25.2	22.4	22.6	1.6	1.8	3.2	3.0
7	Pariet (rt)	0.2 / 0.1	4	5	24.3	25.6	22.7	24.5	2.8	2.3	4.8	5.1
8	Pariet (rt)	0.4 / 0.5	4	4	24.4	26.1	23.2	23.4	2.5	2.0	4.0	3.6
9	Front (rt)	0.3 / 0.2	4	4	23.3	26.4	22.5	24.0	2.2	2.7	4.2	4.5
	mean	0.5 / 0.5	4	4.6	23.6	25.8	22.6	24.0	2.12	2.16	3.88	4.08
		p=0.69			p <0.001		p <0.001		p=0.60		p=0.51	

**Table 4:** Comparison on OAR sparing between iPlan-MIDCA and ABMP-SIDCA (Case 2) Prescription: PTV D95%=100% dose (22 Gy) OPN-OPT=optic nerve-optic tract, rt=right, lt=left

	Max. dose (Gy)	
	iPlan	ABMP
Brainstem	9.5	8.8
Eye (lt)	0.5	2.0
Eye (rt)	0.6	3.2
Lens (lt)	0.3	1.2
Lens (Rt)	0.3	1.5
OPN-OPT (lt)	1.1	3.2
OPN-OPT (rt)	2.0	3.9

### Comparison between ABMP-SIDCA and GKRS

Table 5 showed the comparison between GKRS and ABMP-SIDCA in Case 1. The volume of GTV in GKRS was close to that of no margin-PTV in ABMP-SIDCA. However, the volume of PIV in GKRS was close to that of 1 mm-margin-PTV in ABMP-SIDCA. V (1/2 prescription dose) in GKRS was significantly smaller than that of 1 mm margin V(1/2 prescription dose) in ABMP-SIDCA (p=0.007) but in GI (V (1/2 prescription dose) / V (prescription dose)) was not different (4.74 and 5.26, respectively in mean, p=0.34). This showed that the same level of dose fall-off around the target was obtained in ABMP-SIDCA, as compared with GKRS.

**Table 5:** Comparison between GKRS and ABMP-SIDCA (Case 1) Prescription: PTV D95%=100% dose (20 Gy), PTV= planned target volume, GTV=gross tumor volume, Vol.=volume Cbll=cerebellar, Temp=temporal, Pariet=parietal, Front=frontal, rt=right, lt=left, v.=vermis *In GK, non-intentional margin is added to cover the whole tumor included in the prescription isodose.

	Tumor	GTV (ml)		PTV (ml)		V[Prescription Dose] (ml)				V[1/2PresD] (ml)			
				ABMP		GK	ABMP			GK	ABMP		
	Location	GK	ABMP	Margin 1mm	Margin 2mm	No margin*	No margin	Margin 1mm	Margin 2mm	No margin*	No margin	Margin 1mm	Margin 2mm
1	Cbll (rt)	0.08	0.07	0.19	0.42	0.21	0.11	0.32	0.56	0.80	0.66	1.55	2.58
2	Cbll (lt)	0.04	0.04	0.14	0.32	0.21	0.07	0.17	0.49	0.80	0.46	0.97	2.10
3	Cbll (lt)	0.10	0.11	0.28	0.58	0.32	0.17	0.42	1.00	1.00	0.87	1.75	3.51
4	Cbll (v.)	0.01	0.02	0.07	0.2	0.09	0.02	0.12	0.35	0.71	0.23	0.66	1.53
5	Cbll (v.)	0.02	0.02	0.09	0.24	0.10	0.04	0.14	0.34	0.72	0.27	0.78	1.69
6	Temp (rt)	0.18	0.16	0.35	0.68	0.37	0.24	0.58	1.13	1.03	1.19	2.27	3.84
7	Pariet (rt)	0.22	0.11	0.27	0.55	0.48	0.18	0.43	0.91	1.23	0.97	2.01	3.61
8	Front (lt)	0.03	0.03	0.10	0.26	0.10	0.05	0.15	0.54	0.56	0.36	1.05	1.68
9	Front (lt)	0.04	0.03	0.11	0.27	0.17	0.05	0.22	1.26	1.00	0.35	1.32	5.19
	mean	0.08	0.06	0.18	0.39	0.23	0.10	0.28	0.73	0.87	0.60	1.37	2.86
		p=0.28				p=0.001				p=0.008			
		p <0.001				p=0.07				p=0.007			
		p <0.001				p <0.001				p <0.001			

Table 6 showed the comparison between GKRS and ABMP-SIDCA in Case 2. The volume of GTV in GKRS was close to no margin-PTV in ABMP-SIDCA. In addition, the volume of PIV in GKRS was close to no margin-PTV in ABMP-SIDCA. V (1/2 prescription dose) in GKRS was not different with that of no margin V (1/2 prescription dose) in ABMP-SIDCA (p=0.17). This showed that the same level of dose fall-off around the target was obtained in ABMP-SIDCA, as compared with GKRS in Case 2, just like in Case 1. 

**Table 6:** Comparison between GKRS and ABMP-SIDCA (Case 2) Prescription: PTV D95%=100% dose (20 Gy), PTV= planned target volume, GTV=gross tumor volume, Vol.=volume Cbll=cerebellar, Occip=occipital, Temp=temporal, Front=frontal, Pariet=parietal, rt=right, lt=left, v.=vermis *In GK, non-intentional margin is added to cover the whole tumor included in the prescription isodose.

	Tumor	GTV (ml)		PTV (ml)		V[Prescription Dose] (ml)				V[1/2PresD] (ml)			
				ABMP		GK	ABMP			GK	ABMP		
	Location	GK	ABMP	Margin 1mm	Margin 2mm	No margin*	No margin	Margin 1mm	Margin 2mm	No margin*	No margin	Margin 1mm	Margin 2mm
1	Cbll (v.)	0.08	0.12	0.25	0.58	0.28	0.27	0.49	0.84	0.89	1.07	1.84	3.23
2	Occip (lt)	0.06	0.07	0.16	0.42	0.27	0.15	0.29	0.76	0.86	0.78	1.66	3.09
3	Temp (rt)	0.21	0.34	0.58	1.13	0.49	0.58	1.02	1.84	1.32	1.97	3.41	6.08
4	Temp (rt)	0.02	0.04	0.09	0.29	0.17	0.08	0.20	0.54	0.73	0.39	0.87	2.00
5	Occip (rt)	0.11	0.18	0.34	0.75	0.38	0.43	0.67	1.41	1.08	1.60	2.27	4.86
6	Front (lt)	1.05	1.49	2.08	3.32	1.79	2.18	3.38	5.13	5.06	6.70	10.05	17.21
7	Pariet (rt)	0.01	0.03	0.07	0.24	0.17	0.05	0.15	0.36	0.79	0.32	0.75	1.72
8	Pariet (rt)	0.22	0.26	0.46	0.94	0.55	0.44	0.81	1.48	1.46	1.61	2.91	5.34
9	Front (rt)	0.07	0.12	0.24	0.58	0.23	0.29	0.57	1.18	0.83	1.47	2.57	4.83
	mean	0.21	0.29	0.47	0.92	0.48	0.50	0.84	1.50	1.45	1.77	2.93	5.37
		p=0.079				p=0.78				p=0.17			
		p=0.027				p=0.060				p=0.018			
		p=0.009				p=0.011				p=0.008			

### Treatment time

Beam-on time was 270 seconds and the estimated time for the total procedure for all nine tumors simultaneously was about 20 minutes by ABMP-SIDCA in both cases. The corresponding times of iPlan-MIDCA were 240 seconds and about 20 minutes on each of the three days in both cases. Beam-on time of GKRS was 76 minutes in Case 1 and 88 minutes in Case 2.

## Discussion

The effectiveness of GKRS for multiple small brain metastases has been reported repeatedly [1-3]. The need for SRS/SRT for brain lesions is expanding, since recently it is expected that an increasing number of primary cancer lesions will be controllable for long periods. LINAC-based SRS/SRT is also reported to be effective for brain metastases with a small number of tumors [7]. ABMP is a newly developed treatment planning system for multiple brain metastases. In this study, both CI and GI in ABMP-SIDCA were good as compared with conventional iPlan-MIDCA. In GKRS planning procedures, only GTV is usually contoured and the PTV margin is not defined in most cases, especially for round well-contrast-enhanced and well-demarcated metastatic tumors. However, when an isocenter is placed for a lesion, some intentional margin (maybe 1 mm or less), which is not defined as PTV margin, is usually added for the PIV around the GTV. If the lesion is very small like those of the present cases, PIV/GTV tends to be large in GK, because this additional margin for the PIV around the GTV would be relatively large against the small volume of GTV.

In this study, contouring of GTV and definition of PTV, or decision of PTV margin, was also evaluated in ABMP as compared with GK. The PIV of GKRS was very small, or non-defined PTV margin was minimal (1 mm or less). Not only precise GTV contouring but also deciding the optimal PTV margin is very important in SRS/SRT. Only evaluation of CI and GI would not be sufficient, as PTV itself might be larger in SRS plans by LINAC systems. If the radiotherapy system is deficient in accurate targeting and a wide PTV margin is employed, the surrounding normal brain may be damaged by wide diffusion of the radiation dose. To obtain optimal treatment results with less possibility of adverse effects on the surrounding normal brain, the same as by GKRS, efforts at quality control and quality assurance using LINAC systems to reduce possible uncertainties including image distortion, patient setup error and to avoid the need to add large PTV margins are indispensable. In this study, in the comparison between GKRS and ABMP-SIDCA, ABMP-SIDCA provided a good dose fall-off compatible with GKRS, if the minimal PTV margin, less than 1 mm, (1 mm in Case 1 and 0 in Case 2) was adopted.

Recently, various reports have focused on VMAT (volumetric modulated arc radiotherapy) [11-13] for multiple brain metastases. Clark, et al. [11] reported the feasibility of three non-coplanar arc VMAT in an only three metastasis (10, 15, and 20 mm diameter lesions) patient scenario (four cases). They gave no margin to GTV, and the prescription was made as the setting of D (GTV 100%) >100% dose. Reverse Paddick’s CI was reported as 1/0.761 and Paddick GI as 4.21–5.22. If the number of lesions is not large, each lesion can be targeted by some part of the micro-multi-leaf collimator without any overlap. Iwai, et al. [12] reported on two plans in a phantom study of a nine-lesion setting, geometrical placement and a 14-tumor clinical scenario. In most of the 14 tumors (0.03–0.71 cu cm) both reversed Paddick’s CIs and Paddick’s GI were quite large (only graphs are shown). Lau, et al. [13] reported clinical results in 15 patients (total 62 tumors, 2–13 tumors per patient). They showed V (12 Gy) and V (4.5 Gy) of PTV (no margin or 1 mm PTV margin) in the setting of prescription of D95%=100% dose of 20 Gy. V (12Gy) / PTV is quite large, though reverse Paddick CI is good (small). VMAT cannot achieve a good dose distribution if the number of targets is large, such as 10. In VMAT when the number of targets increases, tumor overlap in the collimator leaf direction and an increase in the maximum distance between scattered tumor lesions in the leaf movement direction may occur, and the limitations of each of the machines may make it impossible to cover all of the lesions. ABMP overcame these problems by dividing lesions into two groups, with ‘go’ arc and ‘return’ one, when lesions overlap or are separate in the collimator movement direction. 

In this study, only planning simulation was investigated in ABMP. In our institution (Aichi Medical University) Varian STx (Varian Medical systems, Tokyo) with ExacTrac system (BrainLAB, Tokyo) is used for SRS. The field of multi-leaf collimator is 40 x 20 cm and the maximum opening width of the collimator is 30 cm. The width of micro multi-leafs varies, namely 2.5 mm in the central portion and 5 mm in the peripheral. A PTV margin of 1 mm is thought to be reasonable in our systems. As the next step, evaluation of ABMP with real dose measurement will be needed.

## Conclusions

The conformity and dose gradient with ABMP-SIDCA were as good as those of conventional MIDCA with multiple groups of DCA by each lesion. If the conditions of the LINAC system permit a minimal PTV margin (1 mm or less), ABMP-SIDCA might provide an excellent dose fall-off compatible with GKRS and enable a short treatment time. This study investigated only simulation planning of ABMP. Next, ABMP with real dose measurements will also need to be evaluated.
